# Simplified lung ultrasound protocol shows excellent prediction of extravascular lung water in ventilated intensive care patients

**DOI:** 10.1186/s13054-015-0756-5

**Published:** 2015-02-06

**Authors:** Philipp Enghard, Sibylle Rademacher, Jens Nee, Dietrich Hasper, Ulrike Engert, Achim Jörres, Jan M Kruse

**Affiliations:** Medizinische Klinik mit Schwerpunkt Nephrologie und Internistische Intensivemdizin, Charité-Universitätsmedizin Berlin, Augustenburger Platz 1, 13353 Berlin, Germany; Abteilung für Radiologie, Charité Universitätsmedizin Berlin, Augstenburger Platz 1, 13353 Berlin, Germany

## Abstract

**Introduction:**

Ultrasound of the lung and quantification of B lines was recently introduced as a novel tool to detect overhydration. In the present study, we aimed to evaluate a four-region protocol of lung ultrasound to determine the pulmonary fluid status in ventilated patients in the intensive care unit.

**Methods:**

Fifty patients underwent both lung ultrasound and transpulmonary thermodilution measurement with the PiCCO system. An ultrasound score based on number of single and confluent B lines per intercostal space was used to quantify pulmonary overhydration. To check for reproducibility, two different intensivists who were blinded as to the ultrasound pictures reassessed and classified them using the same scoring system. The results were compared with those obtained using other methods of evaluating hydration status, including extravascular lung water index (EVLWI) and intrathoracic blood volume index calculated with data from transpulmonary thermodilution measurements. Moreover, chest radiographs were assessed regarding signs of pulmonary overhydration and categorized based on a numeric rating scale.

**Results:**

Lung water assessment by ultrasound using a simplified protocol showed excellent correlation with EVLWI over a broad range of lung hydration grades and ventilator settings. Correlation of chest radiography and EVLWI was less accurate. No correlation whatsoever was found with central venous pressure measurement.

**Conclusion:**

Lung ultrasound is a useful, non-invasive tool in predicting hydration status in mechanically ventilated patients. The four-region protocol that we used is time-saving, correlates well with transpulmonary thermodilution measurements and performs markedly better than chest radiography.

## Introduction

Ultrasound is readily available at the bedside and is non-invasive, making it an ideal diagnostic tool in the hand of the intensivist. The detection of B lines by ultrasound of the lung to diagnose pulmonary edema in the setting of emergency medicine has previously been reported [1]. Changes in pulmonary hydration status before and after hemodialysis were detectable using ultrasound [2]. B lines can be described as vertical, narrow-based artefacts spreading up to the edge of the screen. In previous studies, researchers found B lines to be a surrogate of acute interstitial syndrome and confluent B lines to correspond to alveolar edema [3]. In animal studies, a good correlation between lung ultrasound and lung water assessment using gravimetry was found [4]. A steep learning curve has been reported for lung ultrasound, making it a promising tool for the intensivist [5]. Various protocols have been used to assess extravascular lung water (EVLW) in patients in the intensive care unit (ICU) and in outpatients, but to date no agreement has been reached about the best protocol to use in the ICU setting [5]. In a consensus conference, a 28-zone protocol was suggested following studies in which a 28-sector approach was applied in a cardiology setting and in patients undergoing hemodialysis [2,6]. In the ICU setting, simplified models with an eight-sector protocol, and even a two-sector protocol, have been evaluated in comparison with pulmonary capillary wedge pressure (PCWP) and EVLW [2,7,8]. In the critical care population, a good prediction of EVLW through ultrasound using an eight-zone protocol has been reported. Additionally, in a study in which a four-zone approach was evaluated in comparison with PCWP, researchers reported promising results [7,9].

Currently, various methods are used to diagnose pulmonary overhydration and guide fluid therapy in the critically ill patient. Transpulmonary thermodilution as a method to measure extravascular lung water index (EVLWI) has become a standard tool in many ICUs, and it has been shown to have a significant correlation to lung gravimetry as the standard *ex vivo* method to assess EVLW [10]. EVLWI was demonstrated to be an independent marker of outcome in acute respiratory distress syndrome (ARDS) and in a population of mixed ICU patients [11,12]. Chest radiography, computed tomography, and measurement of central venous pressure (CVP) or PCWP are also commonly used to gain information about pulmonary water content. Nevertheless, all these methods have their own drawbacks and pitfalls. Exposure to radiation is unavoidable when serial chest radiography is conducted, and ordering chest radiography and waiting for it to be conducted and processed leads to significant delay in decision-making. Transfer to the computed tomography scanner adds the risk of transport of the critically ill patient. Pulmonary artery catheterization and introduction of central venous and arterial lines are invasive procedures that carry their own risks.

At present, little information is available regarding the use of lung ultrasound for EVLW assessment in mechanically ventilated patients in the ICU. In the critical care setting, it is of key importance to receive the necessary information about pulmonary hydration status on the spot to guide further therapy. Lung ultrasound at the bedside is a promising tool to use to achieve this goal, and a simplified approach may be of great value. Here we report the results of 50 ventilated patients who underwent four-sector lung ultrasound and transpulmonary dilution measurements, chest radiography and CVP measurements for comparison of the utility of the different methods for lung water assessment in the ICU.

## Material and methods

### Patients

We enrolled all patients 18 years of age or older who were admitted to our medical ICUs for various diagnoses and underwent lung ultrasound and transpulmonary thermodilution measurements with the PiCCO device (PULSION Medical Systems, Munich, Germany) (Table 1).

**Table 1:** **Clinical features** ^**a**^

**Demographics**	**Data**
Age, yr	62 (21 to 88)
Sex, M/F	29/21
APACHE II score	27 (11 to 47)
Duration of ventilation, hr	343 (23 to 1,836)
EVLWI score	10.0 (5.0 to 31.0)
ITBVI score	941.5 (535.0 to 1,600.0)
PaO_2_/FiO_2_	205.5 (70.0 to 373.0)
Sepsis	17
Pneumonia	6
Acute respiratory distress syndrome	6
Cardiopulmonary resuscitation	6
Acute myocardial infarction	5
Pancreatitis	2
Liver failure	2
Other	6

^a^APACHE II, Acute Physiology and Chronic Health Evaluation II; EVLWI, Extravascular lung water index; ITBVI, Intrathoracic blood volume index; PaO_2_/FiO_2_, Index of arterial partial pressure of oxygen and inspiratory oxygen concentration. Values are reported as medians (minimum to maximum) or counts.

The study was approved by the local ethics committee of Charité Universitätsmedizin Berlin (EA4/005/14). No formal consent from the patients was needed according to the ethics committee decision. The study was conducted according to the principles of the Declaration of Helsinki [13].

### Ultrasound measurements

**Figure 1:**
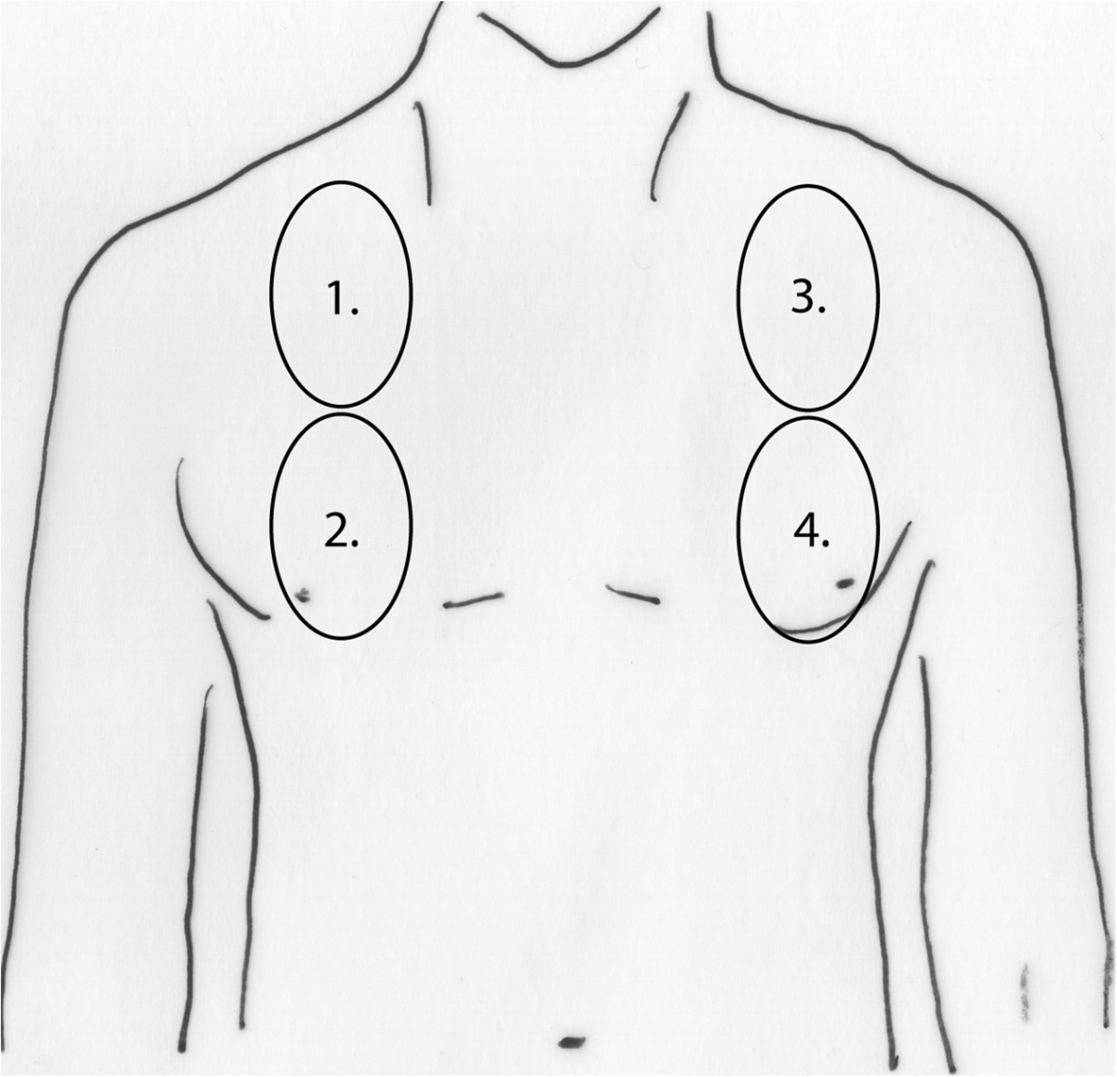
**Scheme of the four parasternal views corresponding to the intercostal spaces between the third and fourth ribs and between the sixth and seventh ribs used to calculate the ultrasound score.**

**Figure 2:**
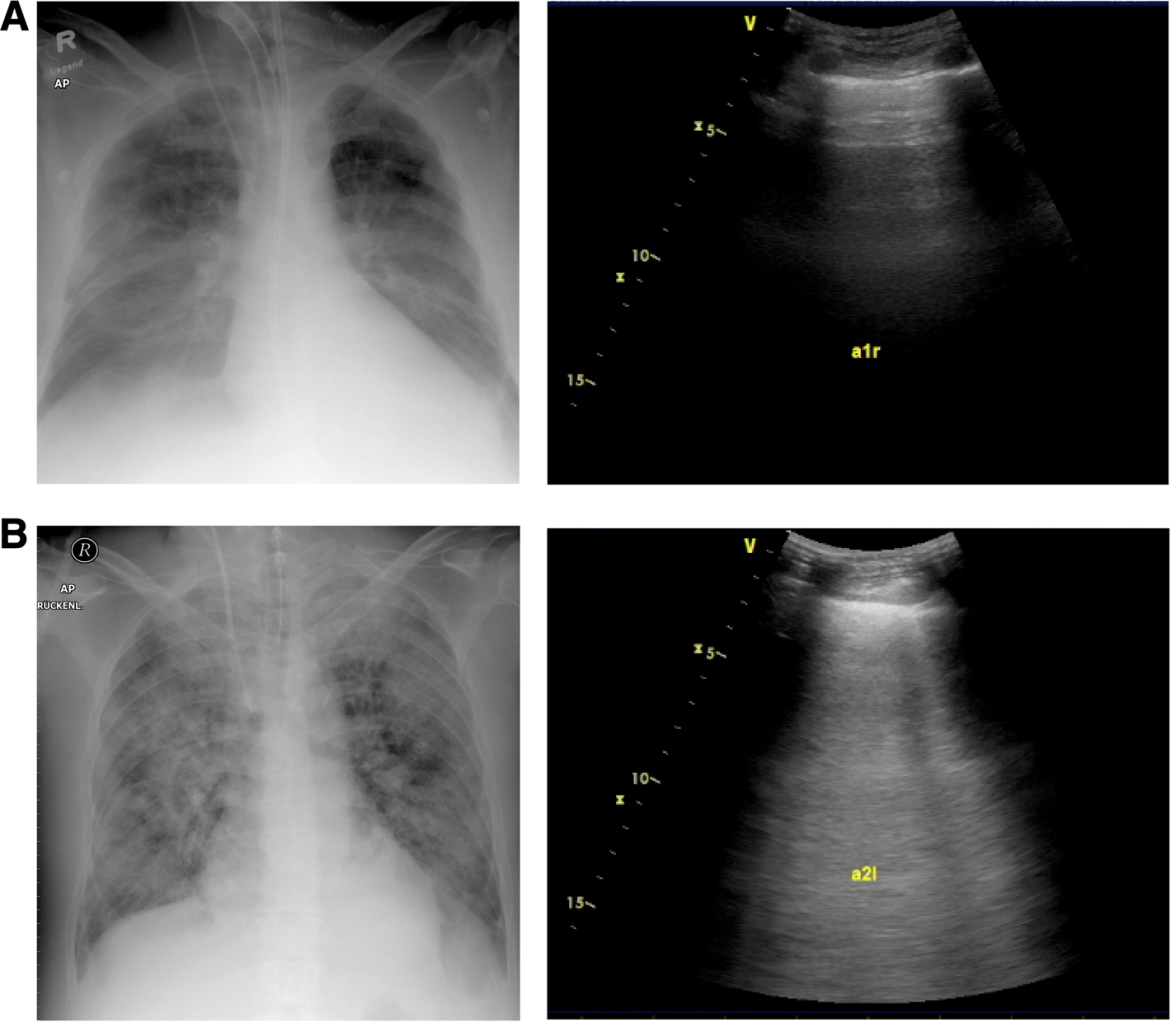
**Chest radiographs (left) and corresponding ultrasound screenshots (right) of two study patients. (A)** Dry lung with a normal extravascular lung water index (EVLWI) and predominant A lines. **(B)** Severe, non-cardiac pulmonary edema with a high EVLWI and confluent B lines.

A Vivid S6 ultrasound machine and a 3.5-MHz curved array probe (GE Healthcare, Chalfont St Giles, UK) were used for all examinations. A single measurement was recorded for each patient. Patients were scanned while in supine position, and four intercostal spaces (ICSs) were examined: the ICS between the third and fourth ribs and the ICS between the sixth and seventh ribs to the left and right of the sternum and between the parasternal and midclavicular line (Figure 1). The number of single and confluent B lines was recorded, and a score ranging from 0 to 32 was calculated to summarize the B lines of the four ICSs (Table 2). Screenshots of every ICS examined were recorded, and two intensivists who were blinded to the details of the images analyzed them using the same scoring system. The averaged result is presented in Figure 2.

**Table 2:** **Ultrasound scoring system**

**Ultrasound finding**	**Score**
No B line/ICS^a^	0
One B line/ICS^a^	1
Two B lines/ICS^a^	2
Three B lines/ICS^a^	3
Four B lines/ICS^a^	4
Five B lines/ICS^a^	5
Confluent B lines >50% ICS^a^	6
Confluent B lines >75% ICS^a^	7
Confluent B lines 100% ICS^a^	8

^a^ICS, Intercostal space.

### Transpulmonary thermodilution measurements

All measurements were performed using the PiCCO device. The PiCCO device was applied only for clinical reasons and independently from the study protocol.

Examinations were performed by application a 20-ml bolus of 0.9% saline at 4°C. At least three single measurements were performed with the patient in supine position. If there was a significant variability in the results, further measurements were performed until three conclusive results were obtained.

### Radiography

Anteroposterior chest radiographs with the patient in supine position were obtained within a 24-hour period before or after the ultrasound measurements were recorded. A senior radiology consultant who was blinded as to their details evaluated them for pulmonary fluid burden using a numeric rating scale ranging from 0 to 32 (low = 0 to 10, moderate = 11 to 20 and high = 21+). Kerley A and B lines, grade and distribution of vascular dilatation and opacities, effusions and cardiac enlargement were assessed.

### Central venous pressure

The central venous catheter had to be placed in either the internal jugular or subclavian vein, and correct position had to be confirmed by chest radiography. Measurements were taken with the patient in supine position after controlling for the correct positioning of the pressure transducer and zeroing of the transducer. Values were taken retrospectively from the patient’s electronic medical record.

### Statistical analysis

The Spearman coefficient was used to determine correlations, and a Bland-Altman plot was generated to check for possible bias. Analysis was performed and graphs were generated using GraphPad Prism 6.0 (GraphPad Software, La Jolla, CA, USA) and SPSS (IBM SPSS, Chicago, IL, USA) software.

## Results

### Presence and extent of B lines intimately correlate with pulmonary water status as assessed by extravascular lung water index

The EVLWI was measured using the PICCO technology and compared with the lung ultrasound findings. The median duration of the lung ultrasound examination was 2 minutes, with a range from 1.5 to 7 minutes. Scanning time was recorded for 40 of 50 patients. All included patients were successfully examined, and no dropouts caused by poor examination conditions occurred.

**Figure 3:**
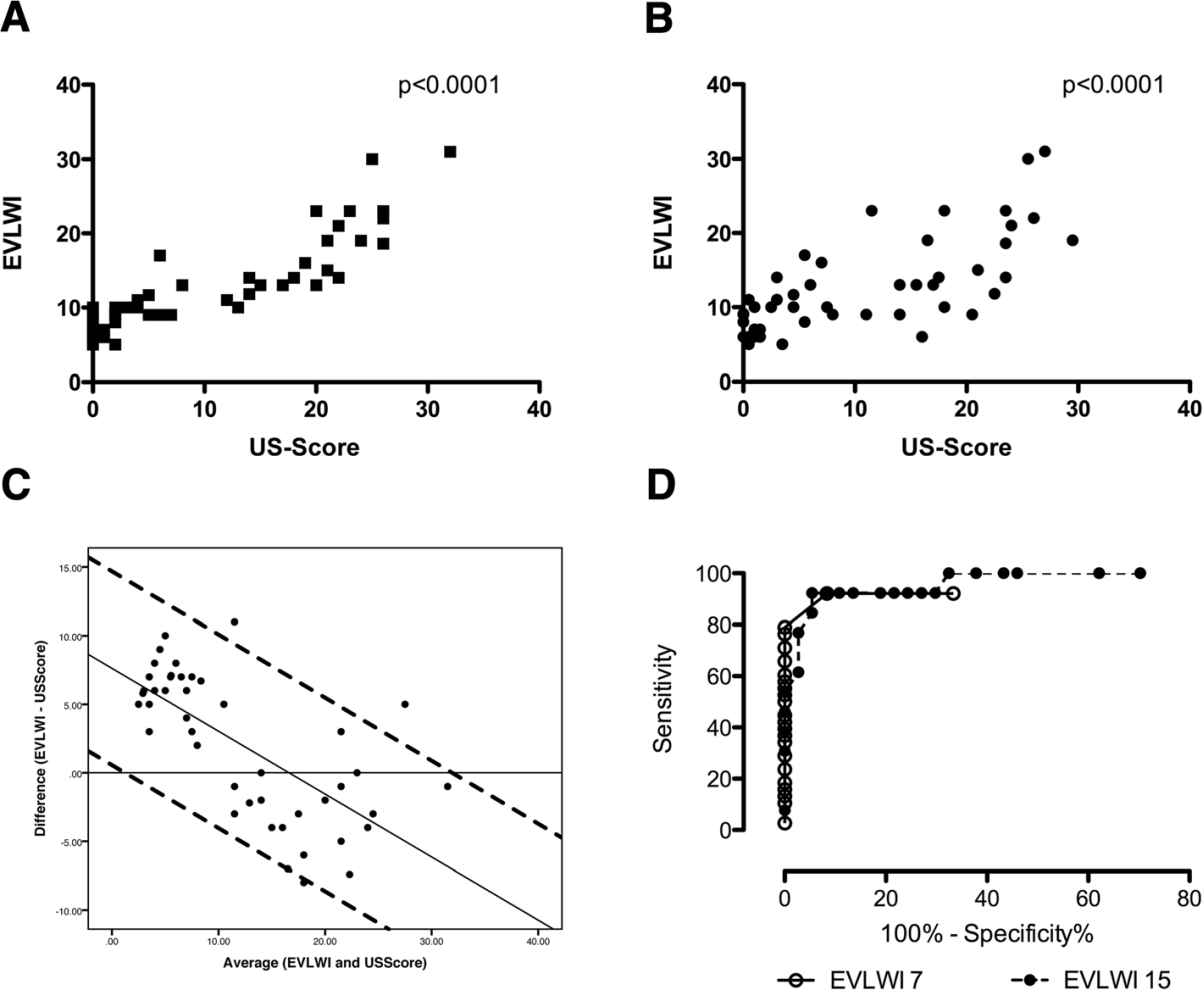
**Correlation of the extravascular lung water index with the ultrasound score. (A)** We found a close correlation of the ultrasound (US) score with the extravascular lung water index (EVLWI) (Spearman’s *r* = 0.91, *P* < 0.0001). **(B)** Correlation of the blinded US score as a mean of two independent examiners is shown (Spearman’s *r* = 0.72, *P* < 0.0001). **(C)** Bland-Altman plot comparing the difference (EVLWI − US score) with the average (of EVLWI and US score). Additionally, a linear regression (difference = 7.62 − 0.46 × average) and the 95% confident intervals (linear regression ± 1.96 × 3.6) are plotted. **(D)** Receiver operating characteristic curves of the US score obtained to identify patients with EVLWIs >7 and >15 show excellent diagnostic performance, as indicated by the areas under the curve of 0.9419 and 0.9636.

The ultrasound score (US score) calculated directly by the examiner performing the examination closely correlated with the EVLWI (Spearman’s *r* = 0.91, *P* < 0.0001) (Figure 3A). To further validate B lines as a tool for assessing the lung water status, the recorded ultrasound pictures were reanalyzed in a blinded fashion by two independent examiners, and the results were averaged. Retrospective blinded assessment slightly reduced the strength of the association with EVLWI; nevertheless, the correlation remained tight and highly significant (Spearman’s *r* = 0.72, *P* < 0.0001) (Figure 3B).

A Bland-Altman plot was calculated to assess for any potential bias by comparing the EVLWI and the US score. A bias of 2.52 (mean difference of EVLWI − US score) was observed. Additionally, the difference and average were not independent, suggesting that in patients with low fluid status, the EVLWI was relatively higher than the US score, and the converse was true with increasing lung fluid. A linear regression was calculated according to the method of Bland and Altman [14]. The linear function- and linear regression-based 95% limits of agreement are shown in Figure 3C.

A receiver operating characteristic curve was calculated to further specify the diagnostic potential of B lines. A US score >1.5 had a sensitivity and specificity of 92.1% and 91.7%, respectively, for diagnosing an EVLWI above the normal value of 7 ml/kg (area under the curve (AUC) = 0.9419). To identify patients with a severely increased EVLWI >15, a US score of >18.5 had a sensitivity of 92.3% and specificity of 94.6% (AUC = 0.9636) (Figure 3D).

### Correlation of ultrasound score and PaO_2_/FiO_2_, central venous pressure and intrathoracic blood volume index

The data indicated a significant but weak correlation between the US score and the index of arterial partial pressure of oxygen and inspiratory oxygen concentration (PaO_2_/FiO_2_) (Spearman’s *r* = −0.34, *P* = 0.02). The correlation between the EVLWI and the PaO_2_/FiO_2_ was also weak, but it was significant (Spearman’s *r* = −0.37, *P* 0.01) (data not shown). Neither CVP (Spearman’s *r* = −0.011, *P* = 0.4924) nor intrathoracic blood volume index (ITBVI) (Spearman’s *r* = 0.16, *P* = 0.2873) was significantly correlated with the presence and extent of pulmonary B lines (data not shown).

### Comparison of chest radiography and central venous pressure with EVLWI and ITBVI

**Figure 4:**
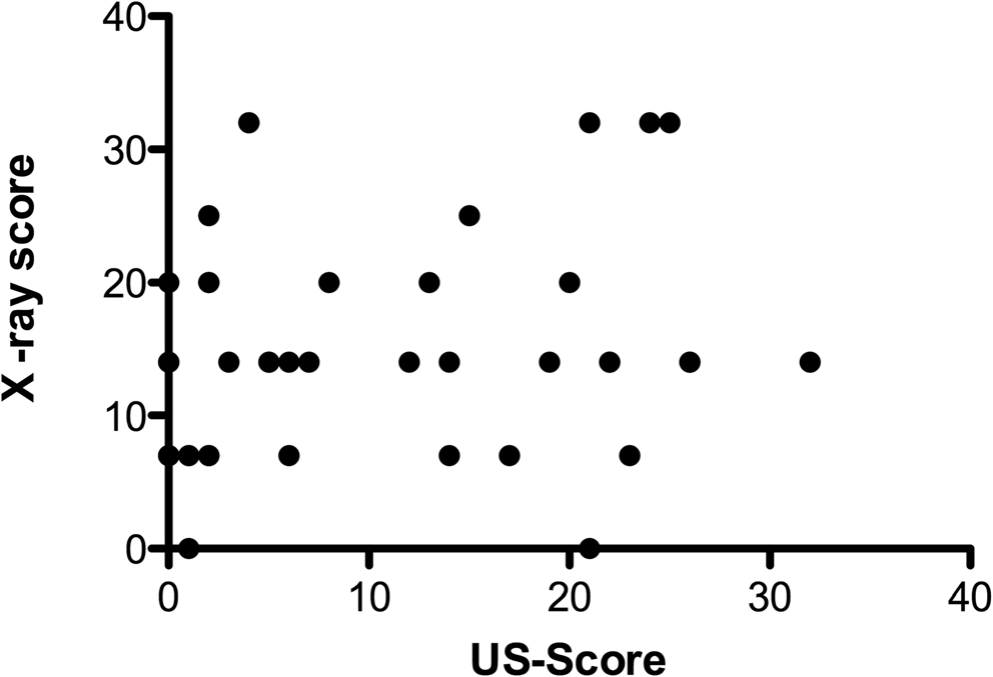
**Comparison of pulmonary fluid status evaluated by chest radiography and ultrasound.** US, Ultrasound.

Chest radiography and EVLWI showed a significant, but rather weak, correlation, with a Spearman coefficient of 0.33 and a *P*-value of 0.03. No significant correlation was found between chest radiography and ITBVI, CVP and PaO_2_/FiO_2_ (Figure 4).

**Figure 5:**
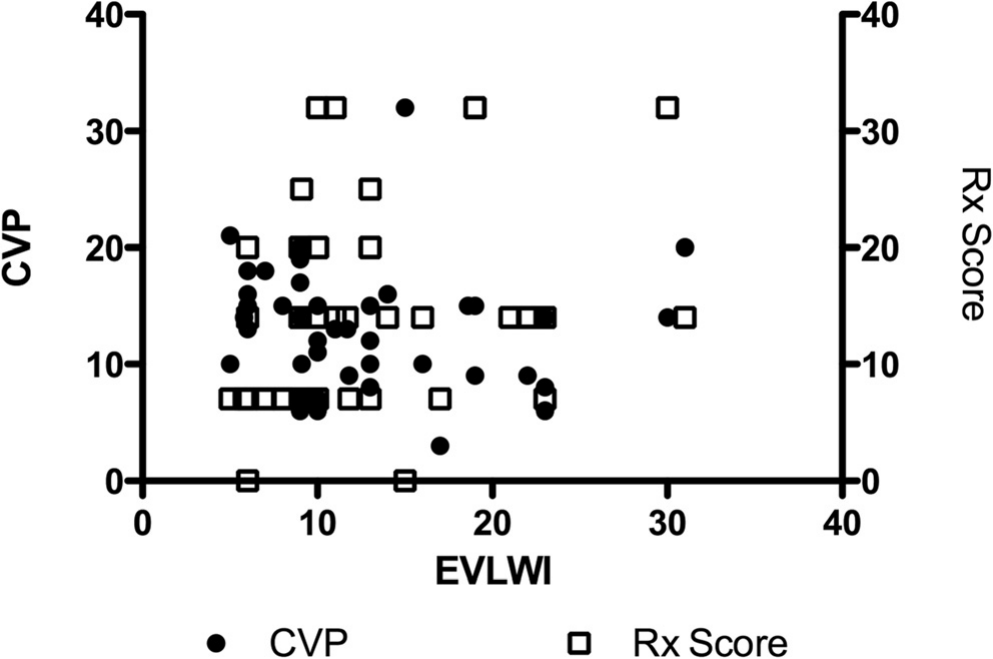
**Correlation of extravascular lung water index to chest radiography (Spearman’s** ***r*** **= 0.33, and** ***P*** **= 0.03) and central venous pressure (Spearman’s** ***r*** **= 0.24,** ***P*** **= 0.11).** CVP, Central venous pressure; EVLWI, Extravascular lung water index; Rx, Chest radiography.

Likewise, no significant correlation was found between CVP and the EVLWI) (Spearman’s *r* = −0.24, *P* = 0.11. Interestingly, there was also no correlation between CVP and ITBVI (Spearman’s *r* = 0.06, *P* = 0.73) (Figure 5 and data not shown).

## Discussion

Our data suggest that lung ultrasound may be a valuable tool in assessing EVLW in patients in the ICU. Our four-sector protocol showed a tight and significant correlation with EVLWI values derived from transpulmonary thermodilution measurements. It had good sensitivity and specificity to exclude clinically relevant accumulation of EVLW and for diagnosis of severe pulmonary edema. Retrospective analysis of the screenshots by different investigators revealed a good correlation with the EVLWI. Simplified lung ultrasound performed markedly better than chest radiography for prediction of EVLWI. The ultrasound examination was easy, noninvasive and fast, making it an attractive approach for assessing pulmonary fluid status.

EVLW accumulation is a common problem in the critical ill patient in general and especially in patients with sepsis and ARDS. It is still debated which diagnostic tool is the best to use for guiding fluid therapy in regard to EVLW in the ICU. To this end, transpulmonary thermodilution or double-indicator measurement, analysis of chest radiography, CVP and pulmonary artery occlusion pressure measurement are used in different institutions [15,16]. Chest radiography is frequently used to assess EVLW, but usually its interpretation is subjective; in addition, the sensitivity and specificity of scoring systems are largely uncertain [16,17]. Measurement of CVP or the use of pulmonary artery catheters is still common, although their utility and value in guiding fluid therapy have been questioned in recent years [18,19]. The measurement of the EVLWI by transpulmonary thermodilution or a transpulmonary double-indicator (thermo-dye dilution) technique was proved to predict outcome in a general ICU population and patients with severe ARDS [11,12]. This measurement method showed significant correlation with lung gravimetry as the standard *ex vivo* parameter for EVLW [10]. It has been shown that even small changes in EVLW can be detected by using transpulmonary thermodilution [20]. For these reasons, it has become the standard method for assessment of EVLW in many institutions and was used as the reference method in our study. However, placement of a central line and a special arterial catheter is required, generating costs and making it an invasive procedure. Possible pitfalls lie in assessment of patients with focal lung injury and vascular obstruction [21].

Using lung ultrasound to detect so-called B lines proved to be a useful diagnostic tool in diagnosing pulmonary edema in the emergency medicine setting and in animal studies, where the detection and quantification of so-called B lines showed correlation with clinical assessment, radiologic findings, natriuretic peptides and pulmonary artery occlusion pressure [1,4,9,22-26]. Other conditions that cause an acute interstitial syndrome such as pulmonary fibrosis and interstitial pneumonitis should be ruled out clinically and by assessment of the sonographic appearance of the pleural line [5,27]. Various protocols have been proposed, but, although a 28-sector approach is recommended in a cardiology outpatient setting, no consensus has been reached about the ideal lung ultrasound protocol in the ICU [5]. In our present study, we were able to demonstrate that a four-sector approach provides similar accuracy in predicting EVLW compared with more complex protocols and might be of value because rapid decision-making is key in the emergency and ICU setting.

Because blinding of the ultrasound examiner to the appearance and clinical volume status of the patient was hardly possible, we recorded screenshots of the ultrasound examination and asked two independent examiners to reassess the US score in a blinded manner. Correlation with EVLWI remained significant as a surrogate for good reproducibility. We believe that the correlation coefficient was slightly lower in the blinded analysis because static screenshots were analyzed, whereas the dynamic real-time examination would be more sensitive in detecting B lines that move with pleural sliding images. Nevertheless, only an examination by two independent operators within a narrow time window would prove good reproducibility. This is clearly a limitation of our study.

Correlation of US score and EVLWI with the PaO_2_/FiO_2_ was significant but rather weak. This is in agreement with earlier findings [11] and is not unexpected, given the fact that fluid overload is only one of many factors influencing the pulmonary gas exchange. Assessment of the chest radiographs using a numeric scale revealed a significant but weak correlation with the EVLWI. Given the fact that the correlation of the chest radiographs to the EVLWI was worse than that of the US score, and keeping in mind the risks to the patient associated with radiation exposure due to repeated radiologic examinations and the fact that chest radiographs are not always readily available at the bedside, we conclude that the ultrasound examination might be a better way to conduct an EVLW assessment in the ICU. Researchers in previous studies also reported conflicting results regarding the performance of chest radiographs in predicting pulmonary hyperhydration, interstitial syndrome or high cardiac filling pressures [28-30]. Our data suggest that lung ultrasound is a valuable method to use for assessing EVLW at the bedside of the ventilated ICU patient.

One of the major limitations of our study is the fact that it was done at a single center. We did not compare different protocols using, for example, an 8- or even a 28-zone approach, so no final conclusions can be drawn regarding the superiority of either protocol. We defined transpulmonary thermodilution as our standard method. No consensus has been reached so far regarding the threshold for a pathologic EVLW level. The cutoff values of 7 and 15 ml/kg used in our study were chosen on the basis of different reported mortality rates in critically ill patients associated with these values, but they remain arbitrary [12]. Nevertheless, the results of using lung ultrasound as a bedside tool in the ICU are promising and should prompt further studies to evaluate its utility for making diagnoses and guiding therapy.

## Conclusions

Assessment of EVLW by lung ultrasound using a simplified four-sector protocol shows excellent correlation with the results of transpulmonary thermodilution. The performance of lung ultrasound appears to be superior to chest radiography. The measurement of CVP does not reliably predict pulmonary hydration status in this setting.

## Key messages

- Ultrasound assessment of pulmonary fluid status can be performed by following a simplified protocol that allows rapid decision-making in the critical ill patient.
- A simplified lung ultrasound protocol shows significant correlation to EVLW measured by using a transpulmonary thermodilution technique and performs markedly better than chest radiography.
- Ventilator settings do not significantly influence the accuracy of lung ultrasound assessment of EVLW in the critical ill patient.

## Abbreviations

APACHE II: Acute Physiology and Chronic Health Evaluation II
ARDS: Acute respiratory distress syndrome
AUC: Area under the curve
CVP: Central venous pressure
EVLWI: Extravascular lung water index
ICS: Intercostal space
ICU: Intensive care unit
ITBVI: Intrathoracic blood volume index
PaO_2_/FiO_2_: Index of arterial partial pressure of oxygen and inspiratory oxygen concentration
PCWP: Pulmonary capillary wedge pressure
SD: Standard deviation
US: Ultrasound
